# Editorial: Epilepsy and dementia in stroke survivors

**DOI:** 10.3389/fneur.2023.1320031

**Published:** 2023-11-02

**Authors:** Tomotaka Tanaka, Bibek Gyanwali, Shinya Tomari

**Affiliations:** ^1^Department of Neurology, National Cerebral and Cardiovascular Center, Suita, Japan; ^2^Memory Ageing and Cognition Centre, Yong Loo Lin School of Medicine, National University of Singapore, Singapore, Singapore; ^3^Hunter Medical Research Institute, University of Newcastle, Newcastle, NSW, Australia

**Keywords:** post-stroke epilepsy, vascular dementia, stroke survivor, complication, epilepsy, dementia, superficial siderosis, small vessel diseases

Stroke survivors present with a wide range of neurological, physical, and psychological sequela and complications. Post-stroke epilepsy (PSE) and vascular dementia (VaD), significantly impact both the quality of life and clinical outcomes for these individuals. As advances in acute stroke treatment have led to reduced mortality rates, our aging population has increased the number of stroke survivors, making it crucial to understand and address PSE and VaD.

Approximately 5–10% of stroke survivors suffer from PSE, and identifying the risk factors for PSE is of utmost importance (1). Numerous studies have been conducted in the past, leading to the development of CAVE (2) and SeLECT (3) scores for risk stratification of PSE. Furthermore, it has been reported that status epilepticus in early seizure and cortical superficial siderosis were associated with PSE (4, 5). Costa et al. also highlight the association between hemorrhagic strokes, younger age, and extended hospital stays with PSE in Umbria. Do et al. investigate predictive factors for PSE in stroke survivors under the age of 45. They identify seizures at the first admission of stroke, stroke severity, recurrent strokes, and drug abuse as significant predictors, while statin use is linked to a lower risk of PSE. Other studies have also reported that statins might have a prophylactic effect against PSE (6). The anti-epileptogenic effect might be derived from statin's pleiotropic effects, such as its anti-inflammatory, antioxidant, and anti-thrombotic properties (7). However, rigorous randomized controlled trials are needed to establish conclusive evidence. Moreover, recent advances in genetic testing have led to the identification of specific genes and risk genes associated with various diseases, including PSE. A systematic review found that *TRMP6 rs2274924, ALDH2 rs671*, and *CD40*−*1C/T* were significantly associated (8). Gao et al. also report the genetic factors contributing to PSE by using bioinformatics analysis in subarachnoid hemorrhage (9). Exploring genetic backgrounds and identifying risks of PSE promise to shed further light on the complexities of PSE and play crucial roles in primary prevention research.

Small vessel diseases (SVD), including cerebral microbleeds, white matter hyperintensities, lacunes, and enlargements of the perivascular space, have gained attention as imaging biomarkers in the elderly. These features have been associated with depression, epilepsy, and VaD. Furthermore, since these markers frequently co-occur, composite scoring systems such as the SVD score have become renowned for stratifying the risk of VaD and related conditions (10). However, we still lack a clear understanding of the specific challenges faced by individuals with severe SVD scores. Lin et al. provide valuable insights into factors associated with dementia, particularly the role of subcortical white matter hyperintensities and lacunes in those with multiple cerebral microbleeds. Their findings suggest the potential for personalized treatment for severe SVD cases. Recent advances in amyloid antibody therapy have brought attention to diagnosing states resembling early stages such as prodromal or preclinical dementia. Ji et al. find that 13.7% of participants exhibit cognitive improvement over 1 year after stroke in patients with subjective cognitive complaints. Factors such as gender, baseline MoCA scores, coffee consumption, and thalamus strokes are associated with these improvements. These findings might help identify patients who may benefit from early intervention.

Regarding the prevention of dementia, intensive blood pressure management, aiming for a systolic blood pressure goal of <120 mm Hg, has been reported to reduce the risk of dementia in the SPRINT MIND study (11). Wu et al. reveal that the prevalence of post-stroke dementia among stroke survivors in China is 17.8%. Aside from aging and educational level, poorly controlled blood pressure is an independent risk factor for dementia. However, the optimal blood pressure range for elderly individuals and those with severe extra- or intracranial stenosis remains an important question for future research.

In summary, the interactions between post-stroke complications, including PSE and VaD, remain a critical concern for stroke survivors. The advancement of acute stroke treatments through thrombolysis and endovascular therapies has significantly reduced mortality rates, resulting in a growing population of stroke survivors. Understanding the post-stroke complications, such as PSE and VaD, has now become imperative. The studies discussed offer glimpses into the problems of stroke survivors and leave us hopeful for personalized interventions and a brighter future for stroke survivors.

## Author contributions

TT: Writing—original draft, Writing—review & editing. BG: Writing—review & editing. ST: Writing—review & editing.
